# Local Anesthetics, Procaine, Lidocaine, and Mepivacaine Show Vasodilatation but No Type 1 Allergy: A Double-Blind, Placebo-Controlled Study

**DOI:** 10.1155/2017/9804693

**Published:** 2017-12-11

**Authors:** Stefan Weinschenk, Caroline Mergenthaler, Christina Armstrong, Richard Göllner, Markus W. Hollmann, Thomas Strowitzki

**Affiliations:** ^1^Department of Gynecological Endocrinology and Fertility Disorders, University of Heidelberg, Heidelberg, Germany; ^2^Outpatient Practice Drs. Weinschenk, Scherer and Colleagues, Bahnhofplatz 8, 76137 Karlsruhe, Germany; ^3^Heidelberg University Neural Therapy Education and Research Group (The HUNTER Group), Heidelberg, Germany; ^4^Medical School, University of Heidelberg, Heidelberg, Germany; ^5^Institute of Educational Science, University of Tübingen, Tübingen, Germany; ^6^Department of Anesthesiology AMC Amsterdam, University of Amsterdam, Amsterdam, Netherlands

## Abstract

**Background:**

Therapy with local anesthetics (LAs), also known as neural therapy, is used in integrative medicine because of its anti-inflammatory properties. Ester-linked LAs are often avoided because of their alleged high allergenicity. Little data supports this assumption and hence the importance of our investigation on type-1 allergies against ester- and amide-linked LAs. We performed a prospective, double-blinded, placebo-controlled observational study.

**Methods:**

177 patients received 340 intracutaneous injections with 1% procaine, 0.5% lidocaine, 1% mepivacaine, or saline solution. Every patient received two different tests on each forearm. Reactions were monitored for 15 minutes.

**Results:**

No type-1 allergy was observed. The mean erythema diameter of the wheals after 10 minutes was procaine 8.0 ± 6.4 mm, mepivacaine 7.6 ± 6.3 mm, lidocaine 4.4 ± 4.8 mm, and NaCl 3.7 ± 3.2 mm. The wheal diameter of all substances showed a crescendo-decrescendo phenomenon. The procaine and mepivacaine wheals were significantly larger than those of lidocaine and NaCl. No general signs of hypersensitivity were observed. Diameter and intensity were independent of the injection site, order of injection, age, gender, and body mass index.

**Conclusion:**

This study shows no higher type-1 allergenicity of the ester-linked LA procaine, compared to the amide-linked LAs lidocaine and mepivacaine, and supports its use in therapy and short-track surgery.

## 1. Introduction

In complementary and integrative medicine (CIM) local anesthetics (LAs) are frequently used for the therapy of chronic diseases and complex functional disorders [1]. Such therapy is known as therapy with local anesthetics (TLA) or neural therapy (NT) [2]. Pain physicians frequently use superficial injections of LAs to treat acute and chronic pain [1]. Recent findings on the different molecular properties of LAs also support their therapeutic use in other subspecialties, for example, gastroenterology [3], oncology [4], and other fields.

Given therapeutic indications, physicians often will use procaine, a short-acting ester-linked local anesthetic (ELA). Procaine was the only available LA for more than 40 years; in 1947 the first amide-linked local anesthetic (ALA), lidocaine, was synthesized. Today procaine is still used and achieves long-term therapeutic effects in CIM. Nevertheless many physicians believe that ELAs, such as procaine and chloroprocaine, are highly allergenic. This idea is propagated in numerous textbooks, although it is presumably incorrect. It has caused a widespread avoidance of ELAs, in favor of ALAs. An older investigation reports an allergenicity rate of up to 20% [5]. This alleged high allergenicity was tentatively attributed to a cross-allergy against para-aminobenzoic acid, one of the cleavage products of ELAs [6, 7]. Since Aldrete and Johnson's investigation of some 60 patients, a background prevalence study, with a sufficient and comparable sample size and also about ELA allergy, has not yet been conducted.

A study about the vasoactivity of LAs [8] reported an increased vasodilatation for ELAs, whereas ALAs tend to cause vasoconstriction. Willatts and Reynolds described similar symptoms to those observed by Aldrete and Johnson. Willatts and Reynolds did not consider them an allergic reaction. It is also remarkable that procaine is so widely used, without notable skin responses being reported. Both these considerations speak against a high allergenicity of the respective substances.

Recently, it has been proposed that ALAs also have a higher allergenic potential than previously suspected [7]. Furthermore, certain ALAs cause delayed adverse responses [9], which limit their therapeutic use. In contrast, ELAs are cleaved immediately at the injection site by the local cholinesterases; this explains why ELAs may have fewer adverse effects in the systemic, cardiac, and central nervous systems. Nonetheless it is mainly ELAs, rather than ALAs that are being avoided.

Beside their therapeutic use in integrative medicine, LAs are also used during modern short-track surgery. This is because they are well-controllable and short-acting, which reduces postoperative surveillance times and enables a quick recovery. Using short-acting ELAs could reduce management costs and improve outcomes. Outpatient clinics and emergency units, especially in the United States, prefer to use chloroprocaine for regional anesthesia. However, they do so skeptically, because of ELAs' alleged high allergenicity. The aim of our study was to reevaluate ELA allergy rates compared to that of ALAs and NaCl (placebo) in a large sample of normal-risk patients, that is without a history of adverse reactions to LAs. We compared the skin responses, after an intracutaneous test injection with one of three different LAs (one ELA and two ALAs) or a saline solution. We also intended to investigate any other potential allergic properties of the LAs.

We hypothesized that there would be no significant difference in the rate of allergic reactions between the ELA and ALAs; furthermore the allergenicity would be less than previously suspected. This would restore ELAs' reputation as an important and suitable drug family, while also encouraging new approaches to its therapeutic use in CIM and in outpatient short-track surgery.

## 2. Methods

In a regular healthcare setting, we prospectively collected data on the skin response following an intracutaneous test injection with a LA. The data was collected from patients, who subsequently received their first therapeutic local anesthesia (TLA). We performed 371 intracutaneous injections on 184 individuals. Of these, 340 injections from 177 patients were evaluated. The study was approved by the ethical committee of the University of Heidelberg, with a vote of 487/2011. All patients gave their written informed consent.

### 2.1. Patients

Patient enrollment for the study lasted from January 2, 2008, to March 31, 2014. Final follow-up data was obtained at the end of April 2014. In this period, 612 patients were newly enrolled in the CAM unit of an OB/GYN and pain management practice in Karlsruhe, Germany. In 199 there was an indication for neural therapy. From these, 184 consecutive patients received a test injection at their first appointment of a treatment series. Only patients receiving LAs for the first time were included in the study. All physicians were experienced in TLA/NT.

**Figure 1:**
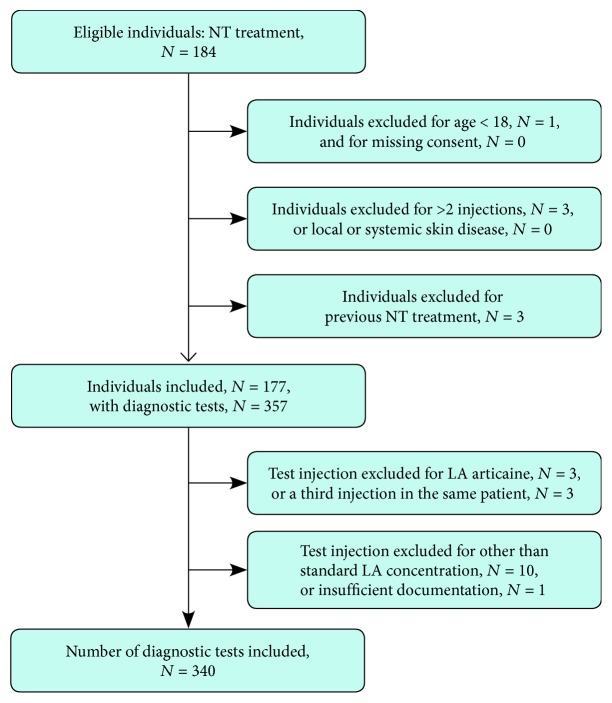
Flow chart of patient selection according to the STARD initiative [10].

Exclusion criteria (Figure 1)are as follows: age < 18 years (*N* = 1 patient); injection with any LA within the last 4 weeks (*N* = 3 patients); more than two intracutaneous test injections at the same time (*N* = 3 patients); generalized or local skin disease at the injection site (*N* = 0).

Out of 184 individuals intended to treat (ITT), 177 were evaluated (96.8%). Every patient received two intracutaneous test injections. Out of 354 injections, 340 were evaluated (96.1%); see Figure 1. Some data had to be excluded from the analysis, because one test used a different LA (articaine, *N* = 3) and 10 injections used a different concentration of procaine or lidocaine. A further test was excluded due to insufficient documentation of responses. In these 14 cases, only the data of the test injection was excluded, that of the patients not. The examined medical history of the patients included their last exposition to LAs and a general allergy anamnesis. We only counted signs and symptoms of an allergic adverse drug reactions (ADR) as a “history of allergy” but no intolerance to animal or herbal products (pollinosis), nor to food or other substances (heavy metals). The biometric data of the patients included their weight, height, and body mass, which were used to calculate their body mass index (BMI). All biometric data is described using the mean ± standard deviation, respectively.

### 2.2. Sample Size Calculation and Statistical Evaluation

We conducted a prestudy power calculation based on the results of the effect sizes given by the only available study of Aldrete and Johnson [5] and the statements in pharmacology standard textbooks that “ester LA (ELA) having a high allergenicity,” which is defined in pharmacovigilance textbooks as an incidence rate of 1–10%.

As these seemed to be an unrealistically high incidence, we conducted a power calculation for three different levels of procaine-induced allergies, that is, 20% [5], 10%, and 5%, a potentially “real” value. For comparison, we chose two incidence rates for amid-LA induced allergy of 0.1% and 1%, respectively, as described in the literature [7].

The power analysis was performed on the basis of a one-tailed binomial test model. Given a 0.05 significance, a 0.80 power, and an ALA allergy incidence of 1%, we estimated a number needed to treat of 14 patients (20%), 29 patients (10%), or 110 patients (5%) required in the procaine group to avoid false negative results. Taken an ALA allergy incidence of 0.1%, the numbers were 8 patients (20%), 16 (10%), and 32 (5% ELA allergy) in the procaine group. For power analysis, these numbers are valid independently from the numbers in the three other groups. Thus, the power achieved in our study with *N* = 177 patients in the procaine group seemed to be adequately high to avoid false negative results.

The data was collected in Excel and transferred into SPSS data sheets (IBM SPSS Inc.), v.21 for statistical analysis. We used a multilevel regression model to compare the skin responses between the groups and the differences in patients' characteristics. The significance level for all tests was set to *p* = 0.05 (two-tailed; multilevel analysis).

### 2.3. LAs, Allocation, Blinding, and Injection Technique

Our protocol remained constant for the whole recruiting period and included a blinded randomization applicable to the every-day use in a normal outpatient clinic. As it was an observational study, following the ethical committee's vote, no change to the usual procedures of this clinic was allowed for the purpose of this study. We used 1% procaine from bottles (Steigerwald Inc., Darmstadt, Germany, and Loges Inc., Winsen/Luhe, Germany) or ampules (Steigerwald Inc.) in 134 cases; 0.5% lidocaine from bottles or ampules (both Steigerwald Inc.) in 96 cases; 1% mepivacaine from ampules or carpules (both Actavis Inc., Hamburg, Germany) in 61 cases; and 0.9% NaCl from bottles (Braun Inc., Melsungen, Germany) in 49 cases; see Table 1. The use of multidose bottles is standard care when LAs are frequently applied in a pain unit. In this investigation, standard precautions for their usage were kept, for example, usage of 22 *μ*m bacteria filters on every bottle, and not using an opened bottle for more than 24 h. Seven different drug preparations from four different manufacturers were available and were used with or without the standard preservatives and adjuvants available in this clinic (Table 1).

Due to the study protocol, the assisting nurse was instructed to randomly select one of these preparations. The allocation and order of the injections were randomized and blinded by numbering each of the solutions. The nurse chose the preparation out of the stock available in the respective room on the respective day for blinding and was prompted not to choose other drugs than those listed above. The 2 ml-syringes were prepared outside of the consulting room by a registered nurse. The two syringes were numbered either as 1 or as 2. The syringes were injected according to their number and without knowing their content (1 = left forearm, 2 = right forearm). Each injection contained 0.1–0.2 ml, which caused an immediate 3-4 mm diameter wheal. The injections were double-blinded, that is, neither the physician administering the injections, nor the patient receiving them knew the content of the syringes. The nurses preparing the solutions did know but were not involved in data collection and evaluation process.

### 2.4. Assessment of Reactions

The skin responses and general symptoms were recorded by the physician after 1, 2, 5, 10, and 15 minutes on a standardized form. For the diameter measurement a millimeter scale was used. The erythema intensity was compared with a color scale (none, light red, and dark red). The itching at the injection site, the appearance of urticaria, bronchospasms, and other allergy signs were described by the patient, using a nominal analogue scale (NAS) from 0–10 after 1, 2, 5, 10, and 15 minutes. This was also documented on a standardized sheet.

All patients were supervised directly by the physician for 15 minutes. Unblinding of the test allocation occurred after completing the documentation sheet. Upon leaving the practice, patients were asked to look out for and document any further reactions that appear within the following days. This was done on an additional documentation sheet, which they could take home.

### 2.5. Multilevel Analysis

The test results are interleaved in two ways: there was a nesting of two injections per patient and five measurements were nested within one injection. For this reason, a multilevel analysis [11] was applied to exclude mutual influences of the data and to avoid the overestimation of effects [12]. A multilevel analysis is also suitable for data with unequal distances, as was the case in this study (the time differences between the evaluation points were 1, 3, and 5 minutes, resp.). It is also robust against missing data.

## 3. Results

### 3.1. Study Population and Comparison of Patients' Characteristics between Different LAs

The average age of the patients at the time of injection was 49.5 ± 13.6 years (18–82 years); the average BMI was 22.9 ± 3.6 kg/m^2^. 90.4% of the patients were female. Provided every patient had received a single intracutaneous test injection we could divide the study population into four groups corresponding to the substances received. Between these groups, the patients' biometric and anamnestic characteristics were compared. They did not show significant differences (see Table 2).

### 3.2. Local Skin Response in Patients: Crescendo-Decrescendo of Erythema Diameter

**Figure 2:**
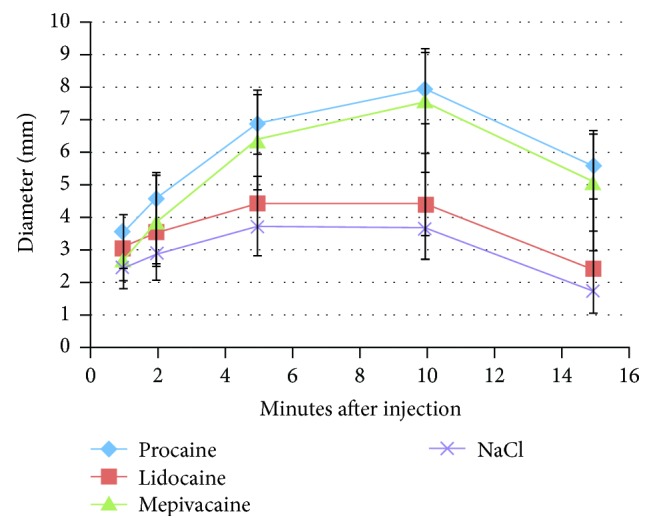
Development of skin reaction within the 340 wheals of 177 patients: diameter of erythema with error bars in millimeters following intracutaneous injection with different LAs and 0.9% saline solution after 1, 2, 5, 10, and 15 minutes, respectively.

Table 3 shows the descriptive results of the erythema diameter in millimeters, for all four groups at 1, 2, 5, 10, and 15 minutes after injection. There was a decrescendo of the diameter between 10 and 15 minutes. In order to provide a more critical examination of the development, a multilevel regression model for all substances was made. The results of the calculation demonstrated a significant parabolic curve character (decrescendo of erythema diameter); see Figure 2.

### 3.3. Intensity of Erythema

**Figure 3:**
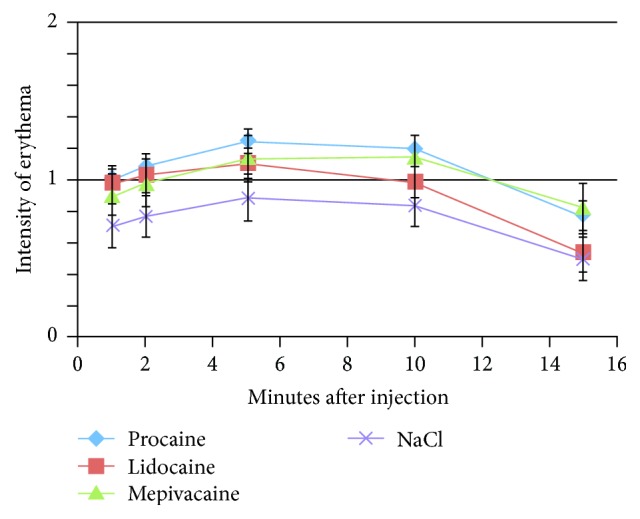
Development of skin reaction: intensity of erythema (0 = none, 1 = light red, and 2 = dark red) with error bars following intracutaneous injection with three different LAs and 0.9% saline solution in 340 tests. Description was documented after 1, 2, 5, 10, and 15 minutes, respectively.

The color of erythema was judged at 1, 2, 5, 10, and 15 minutes after injection by the physician and described as none, light red, or dark red, respectively. Percentages of color intensity for the four substances are listed in Figure 3. It shows the descriptive results of the erythema intensity for all four groups at 1, 2, 5, 10, and 15 minutes after injection. Here a decrescendo of the color intensity between 10 and 15 minutes was also observed. We analyzed the data for all substances with the multilevel regression model. The results of this calculation also demonstrated a significant parabolic curve character of the erythema intensity; see Table 4.

### 3.4. Influence of Covariates

We extended our previous analysis, by including the following covariates: the order of the injections, time-invariants, and biometric factors, such as age, gender, and BMI. The aim was to examine whether or not covariates can predict the initial allergic reaction at 0 minutes and its development with time. To do so, we evaluated the covariates and other important interaction effects again using a multilevel regression model. The results revealed no significant influence of any covariates on the initial response or time-dependent development of the two outcomes: “erythema diameter” and “intensity” (Table 5).

### 3.5. Reports of Allergy Signs

Six patients reported local itching within a 1-2 cm diameter around the injection site, after administration (Table 6). Itching was seen with procaine (*N* = 1), lidocaine (*N* = 2), mepivacaine (*N* = 2), and NaCl (*N* = 2). In none of the patients did these symptoms last longer than 10 minutes. Differences in the distribution of itching between substance groups were not significant.

### 3.6. No Severe Adverse or Allergic Reactions

None of the patients showed signs of systemic allergic or other severe reactions. No conjunctivitis, rhinitis, allergic asthma, larynx edema, or life-threatening symptoms such as an angioneurotic edema (Quincke's edema) or anaphylactic shock were seen in any of the patients. No major bleeding or other severe complications were caused by the injections. The evaluation of the patients' take-home reports at the follow-up appointment revealed that five patients had experienced a possible late (type 4) skin response at the injection sites of mepivacaine (*N* = 2), lidocaine (*N* = 1), and NaCl (*N* = 2).

## 4. Discussion

### 4.1. Allergy or Not?

Ester-linked local anesthetics (ELAs), such as procaine, frequently used local anesthetics (LAs) in integrative medicine; they are used for the complementary therapy of chronic pain. However, their use is often limited by their alleged high allergenicity. This pilot study reevaluates the allergy rate of ELAs in comparison to that of amide-linked LAs (ALAs). It is the first study of its kind. A prospective, double-blinded, controlled study design was used. We found no signs of type 1 or even type 2, or type 3 allergies after intracutaneous injection. There was no significant influence of allergy history or different biometric features on the skin response after injection. We observed a consistent pattern of erythema development, following a square curve for all four substances tested. The skin response, in general, was mild and began to recede after 10 minutes (decrescendo phenomenon). In contrast to this, an allergic reaction of the skin would show a typical crescendo phenomenon and other signs of intolerance.

A type 1 allergy is defined as an IgE-mediated hyperreactivity to an antigen [13]. Its most prominent symptoms are the erythema, skin wheals (“wheal and flare”), and itching. Intracutaneous test injections are considered the most potent triggers of a type 1 allergy. The prick or scratch tests, in comparison, trigger weaker responses. Nevertheless, an erythema, appearing without other signs of an allergy, is not sufficient to diagnose an allergy. Symptoms such as generalized itching, rhinitis, dyspnea, and tachycardia are further clinical signs of an allergy. Most allergy symptoms appear within 10 to 15 minutes after exposure and show a typical crescendo phenomenon [14]; these characteristics are crucial for the diagnosis of an allergy.

ELAs, such as procaine, supposedly possess a high allergenic potential. However, there is little data supporting this assumption and it comes from a select few investigations. In 1970, Aldrete and Johnson observed red skin responses in 60 anesthetized patients. These were seen within a diameter of 1–4 cm around the injection site, up to 120 minutes after intracutaneous injection with procaine, tetracaine, chloroprocaine, lidocaine (lignocaine), mepivacaine, or prilocaine [5]. A “positive skin reaction,” defined as a “wheal with erythema > 1 cm,” was observed only after injection with ELAs and not ALAs. Although no further allergy symptoms like itching, edema, urticaria, or anaphylaxis were observed, the authors concluded that ELAs have a high allergenicity. Another group investigated LA skin reactions in 20 volunteers [15]. Erythema, itching, and pseudopod formation were observed at 8 of the 20 (40%) injection sites for procaine, but not for the other tested LAs. Allergenic potential was not the primary endpoint of this study. That is why these observations were not further investigated.

Subsequent statements about the allergenicity of LAs, in particular those in textbooks, referred to the mentioned studies and were not based on primary data [7]. The difference in erythema induction, between ELAs and ALAs, was explained in theory as a “para-group allergy” [6, 7]. There was no clinical data supporting this idea. Other authors explained the erythema, as a result of vasodilatation [8]. All the while, the alleged high allergenicity of ELAs, such as procaine or chloroprocaine, led to their widespread avoidance in many fields of medicine.

Considering the definition of an allergy it seems that Aldrete and Johnson did not report an allergy, but instead the results of the vasoactivity of LAs [8]. A possible cause of the vasodilatation may be the hydrolytic degradation product of procaine, diethylaminoethanol (DEAE), which has strong vasodilatative properties [16].

In our study, the skin response of the ALA mepivacaine 1% was similar to that of procaine in erythema diameter and color intensity within the first 15 minutes. Lidocaine showed milder skin responses, more similar to those of NaCl. These increased (up to 5 min) and then decreased (until 15 min) in diameter and color intensity for all substances, demonstrating that the reversal of skin response is at least partially independent of the injected substances.

### 4.2. Limitations and Strengths

Our study was based on a single-observer design. No further lab tests, for example, a basophile stimulation test, were performed due to the observational research setting of a daily practice. Moreover, lab tests such as serum-IgE are no longer used during allergy diagnostics [17]. We investigated a homogenic study population and thereby underwent the risk of a potential selection bias; more women than men visit the respective OB/GYN associated practice for integrative medicine. Still, our statistical tests yield a high internal validity. No significant difference between the gender groups was shown. Further studies, with more male subjects, are necessary to be able to fully apply our conclusions to a male population.

The method of blinding and allocation did not meet the highest randomization standards. Because this was an observational study performed in an all-day practice setting, we had to adapt the randomization protocol to the clinical practice in this respective unit in order to meet the rules and regulations of the ethical committee. However, as there were no significant differences between the three LAs and NaCl groups, a post hoc justification of our blinding and allocation method is possible.

The background prevalence of ELA and ALA allergies cannot be derived from our data. To do so, a multicenter and postsurveillance study, with a much larger sample size, would be needed. However, we can conclude that the incidence of ELA-induced allergies is lower than previously assumed. In addition, it does not appear to differ from that of ALA-induced allergies.

Some adverse reactions to LAs were in the past also attributed to the additives in the LA solution [7, 18]. We therefore tested various LA solutions with different or no preservatives. Because no type 1 allergy was seen, evaluating the effect of the different preservatives on the allergenicity of a given LA solution was not possible. Aldrete and Johnson may have used preparations with potentially hazardous preservatives. Unfortunately, they did not report on this important issue in their publication, so today we cannot explain the differences in our results by the absence or presence of additives. Future studies should address this question.

Logistically we could not observe the erythema for more than 15 minutes. The decrescendo phenomenon began after 10 minutes. We therefore concluded that the observation time was most probably sufficient to exclude a further crescendo as a sign of a type 1 allergy.

Because the history of past LA applications was unavailable for some patients, a previous sensitization could not be confirmed or excluded. Furthermore, the test injections themselves could have sensitized our patients. This seems unlikely considering that during further treatment sessions the patients showed no allergy signs, which would be expected in the case of a previous sensitization.

Taking into account the above-mentioned limitations, our conclusion is restricted to the normal population of a daily practice: for such a group, we can conclude that ELAs, such as procaine, show no higher allergenicity than the ALAs, lidocaine and mepivacaine. ELA-allergies also seem to be less frequent than previously assumed.

### 4.3. Need for Short-Acting LAs in Neural Therapy

For most applications in the anesthesiology, ELAs have been avoided since 1970. However, given therapeutic indications, procaine is still used [19], although it is criticized as being highly allergenic.

Nowadays, more and more data on the so-called “alternative effects” of LAs is being published. With the increase in data, a better understanding of the broad spectrum of therapeutic uses of LAs comes, for example, its anti-inflammatory [20] and immunomodulating properties [21], applications in oncology [22, 23], and uses in treating chronic CRPS [24] as well as chronic pain and functional disorders [25]. The therapeutic use of LAs, also known as neural therapy (NT) or therapeutic local anesthesia (TLA), is more relevant than ever [2]. Short-acting ELAs and ALAs seem to be more potent in anti-inflammation and immune regulation, than long-acting LAs [21]. According to our results ELAs are at least equally suited for therapeutic use.

### 4.4. Need for Short-Acting LAs in Modern Short-Track Surgery

Modern short-track surgery also benefits from using shorter-acting LAs. These reduce postoperative surveillance times and enable a quick recovery. Short-acting ELAs are predetermined for time critical interventions, such as an outpatient surgery. Outpatient units in the United States are increasingly using procaine and chloroprocaine, even though these are still considered highly allergenic. In such cases, our results, discrediting the idea of a high allergenicity, are very important to reintroduce and support the clinical use of ELAs.

## 5. Conclusions

An intracutaneous test injection with the ELA, procaine, induces an erythema persisting for more than 10 minutes and is most probably caused by vasodilatation, rather than an allergic reaction. Such a vasodilation is also seen with ALAs.

An erythema, without further symptoms of an allergy, should not be interpreted as an allergy. The 40-year presumption that ELAs cause more allergic reactions than ALAs and generally have a high allergenicity must be questioned.

Furthermore, the ability of LAs to induce vasodilatation not only explains the skin responses but may even contribute to their therapeutic potential in integrative medicine. Our preliminary results show that there is no higher allergy risk when using ELAs instead of other LAs. This risk is not as high as previously suspected. The results endorse the use of procaine in therapy with local anesthetics. Likewise, there are no other major concerns hindering the use of another ELA, chloroprocaine, in short-track surgery and outpatient units. ELAs have their specific indications and can be used. More research is needed to support this statement.

Field studies with larger sample sizes could specify the precise allergenicity rate of ester- and amide-linked LAs so that the prevalence could be determined. This would be a substantial achievement in the field. We encourage such further research, as it betters the use of LAs and fosters good health.

## Figures and Tables

**Table 1:** Drug preparations used in subcutaneous injections for allergy testing of LA.

Substance	Number of injections, *N* =	Container	Preservative	Producer
Procaine 1% *N* = 134	104	Bottle	Benzyl alcohol	Steigerwald
	13	Bottle	Parabens	Loges
	17	Ampoule	None	Steigerwald

Lidocaine 0,5% *N* = 96	82	Bottle	Benzyl alcohol	Steigerwald
	14	Ampoule	None	Steigerwald

Mepivacaine 1% *N* = 61	37	Ampoule	None	Actavis
	24	Carpule	Calcium chloride	Actavis

NaCl 0,9% *N* = 49	49	Bottle	None	Braun

**Table 2:** Characteristics of patients receiving different LAs. LAs used for intracutaneous tests were procaine, lidocaine, mepivacaine, and saline solution (NaCl). Evaluation of 340 injections from 177 patients.

	Procaine 1%	Lidocaine 0.5%	Mepivacaine 1%	NaCl 0.9%	All, *n* = 340	Difference
Female	**90.3**%	**90.6**%	**90.2**%	**87.8**%	**90.2**%	**n.s.**
Age (years)	49.4 ± 13.0	**50.4 ± 13.5**	**49.6 ± 14.5**	48.8 ± 14.4	49.7 ± 13.5	n.s.
BMI (kg/m^2^)	23.0 ± 3.8	**23.0 ± 3.7**	**22.7 ± 3.3**	23.0 ± 3.5	22.9 ± 3.6	n.s.
Previously treated with LA	**98.3**%	**97.7**%	**100**%	**100**%	**98.7**%	**n.s.**
History of drug allergy (antibiotics and others, without LAs)	**10.3**%	**11.4**%	**9.7**%	**9.6**%	**37 (10.9**%**)**	**n.s.**
History of LA allergy	**N** = 0	**N** = 1	**N** = 1	**N** = 2	**N** = 4** (0.12**%**)**	**n.s.**

Allergy history: multiple specifications were possible.

**Table 3:** Diameter of erythema following intracutaneous injection tests in *N* = 177 patients.

Diameter of wheal (mm) with	1 min	2 min	5 min	10 min	15 min
Procaine 1%	3.5 ± 3.4	4.6 ± 4.4	6.9 ± 5.2	8.0 ± 6.4	5.6 ± 6.0
Lidocaine 0.5%	3.1 ± 3.0	3.5 ± 4.8	4.4 ± 4.0	4.4 ± 4.8	2.4 ± 3.0
Mepivacaine 1%	2.8 ± 2.8	3.9 ± 5.4	6.4 ± 5.9	7.6 ± 6.3	5.1 ± 5.8
NaCl	2.5 ± 2.4	2.9 ± 3.1	3.7 ± 3.1	3.7 ± 3.2	1.8 ± 2.6

**Table 4:** Intensity of erythema following intracutaneous injection tests in *N* = 177 patients.

Intensity of erythema with	1 min	2 min	5 min	10 min	15 min
Procaine 1%	1.0 ± 0.4	1.1 ± 0.5	1.3 ± 0.5	1.2 ± 0.5	0.8 ± 0.6
Lidocaine 0.5%	1.0 ± 0.6	1.0 ± 0.5	1.1 ± 0.5	1.0 ± 0.5	0.5 ± 0.6
Mepivacaine 1%	0.9 ± 0.5	1.0 ± 0.6	1.1 ± 0.6	1.2 ± 0.5	0.8 ± 0.6
NaCl	0.7 ± 0.5	0.8 ± 0.5	0.9 ± 0.5	0.8 ± 0.5	0.5 ± 0.5

**Table 5:** Multilevel analysis of estimated effects of covariates, on wheal diameter (dependent covariate). Linear trend (substance): influence of the respective LA on the slope of the time-dependent diameter curve. Quadratic trend (substance): influence of the respective LA on the square shape of the curve. BMI: body mass index. SE: standard error.

	*b*	SE	*p*	95% confidence interval	
				Lower bound	Upper bound
Intercept	2.50	1.07	**0.021**	0.38	4.62
linear trend (all substances)	0.45	0.17	**0.006**	0.13	0.79
Quadratic trend (all substances)	−0.04	0.01	**0.002**	−0.06	−0.01
Procaine	0.01	0.07	0.878	−0.14	0.16
Lidocaine	0.21	0.91	0.815	−1.59	2.01
Mepivacaine	0.00	0.02	0.85	−0.05	0.04
BMI	1.04	0.77	0.18	−0.48	2.55
Gender	0.58	0.82	0.476	−1.02	2.19
Age	0.26	0.83	0.756	−1.37	1.88
Linear trend (procaine)	0.66	0.19	**0.001**	0.27	1.04
Linear trend (lidocaine)	0.04	0.21	0.841	−0.36	0.44
Linear trend (mepivacaine)	0,75	0,22	**0,001**	0,32	1,18
Quadratic trend (procaine)	−0,03	0,01	**0,02**	−0,06	−0,01
Quadratic trend (lidocaine)	0,00	0,01	0,859	−0,03	0,01
Quadratic trend (mepivacaine)	−0,04	0,02	**0,015**	−0,07	−0,01

**Table 6:** Patients with local itching after intracutaneous tests. Six patients reported local effects; all effects receded within 10 minutes.

Patient number	Substance	Observation time: minutes after injection				
		1 min	2 min	5 min	10 min	15 min
095	Procaine	−	−	+	−	−
070	Lidocaine	−	+	+	−	−
161	Lidocaine	−	+	−	−	−
093	Mepivacaine	−	−	−	+	−
165	Mepivacaine	−	+	−	−	−
	NaCl	−	+	−	−	−
163	NaCl	−	+	−	−	−
